# Protocol for assessment of pain biomarkers in orthopaedic patients undergoing total knee arthroplasty in a tertiary care hospital and neuroimmunopharmacology laboratory in South Australia: a cross-sectional matched-subject observational study

**DOI:** 10.1136/bmjopen-2026-117858

**Published:** 2026-06-22

**Authors:** Margot B Aalders, Laura van Marle, Samuel G Evans, D-Yin (Indy) Lin, Christopher Wilson, Johan W Verjans, Job N Doornberg, Mark R Hutchinson, Ruurd L Jaarsma

**Affiliations:** 1 Department of Orthopaedic Surgery, Amsterdam UMC, University of Amsterdam, Amsterdam, The Netherlands; 2 Department of Trauma and Orthopaedic Surgery, Flinders Medical Centre, Flinders Dr, Flinders University, Bedford Park, South Australia, Australia; 3 Department of Orthopaedic Surgery, University Medical Centre Groningen, University of Groningen, Groningen, The Netherlands; 4 School of Biomedicine, The University of Adelaide Faculty of Health and Medical Sciences, Adelaide, South Australia, Australia; 5 Department of Anaesthesia and Perioperative Care, Flinders Medical Centre, Flinders Dr, Flinders University, Bedford Park, South Australia, Australia; 6 Division of Research and Innovation, Australian Institute for Machine Learning (AIML), University of Adelaide Press, Adelaide, South Australia, Australia; 7 (SAHMRI), South Australian Health and Medical Research Institute Limited, Adelaide, South Australia, Australia; 8 Institute for Photonics and Advanced Sensing (IPAS), University of Adelaide, Adelaide, South Australia, Australia

**Keywords:** MENTAL HEALTH, Knee, PAIN MANAGEMENT, Chronic Pain, IMMUNOLOGY

## Abstract

**Introduction:**

Knee osteoarthritis is a leading cause of pain and disability and frequently results in total knee arthroplasty (TKA). Decisions about surgery and postoperative management rely largely on subjective pain scales and patient-reported outcome measures (PROMs), and 10–15% of patients remain dissatisfied after TKA despite technically successful surgery. Evidence suggests that pain is partly reflected in peripheral immune signalling, yet this neuroimmune interface has not been studied in patients with joint pain due to knee osteoarthritis. This study will explore whether peripheral immune responses (interleukin-1 beta, IL-1β) are associated with pain and may serve as objective pain biomarkers in patients with painful knee osteoarthritis compared with pain-free controls and how these markers relate to pain and psychological (anxiety, depression and pain catastrophising) PROMs. This will be the first study to correlate pain markers (peripheral immune responses) with subjective pain levels in orthopaedic patients with symptomatic knee osteoarthritis.

**Methods and analysis:**

This is a protocol for a prospective cross-sectional matched-subject observational study. We will include 20 adults undergoing unilateral primary TKA for painful advanced knee osteoarthritis, their contralateral pain-free knees as internal controls and 20 age and sex matched healthy controls without joint pain or functional limitation. All participants will undergo standardised clinical assessment and complete pain and psychological PROMs. In patients with TKA, venous blood will be collected pre-operatively. During TKA surgery, synovial fluid will be aspirated from the painful operated knee and the contralateral pain-free knee. Healthy controls will provide a single venous blood sample at a hospital visit. Peripheral blood mononuclear cells and synovial fluid mononuclear cells will be stimulated ex vivo with toll-like receptor 2 and 4 agonists to quantify IL-1β release. In parallel, hyperspectral imaging will characterise unstimulated immune cell phenotypes. Multivariable statistical and machine-learning approaches will relate biomarker profiles to pain and psychological PROMs.

**Ethics and dissemination:**

The study has been approved by the Southern Adelaide Local Health Network (SALHN) HREC (references: 2024/HRE00253, SSA 2024/SSA00641). Written informed consent will be obtained from all participants. Study results will be disseminated through peer-reviewed publications and presentations at national and international scientific conferences.

**Trial number registration:**

ACTRN12626000084381.

**STRENGTHS AND LIMITATIONS OF THIS STUDY**

- Integration of immune signatures with subjective pain and psychological health, and inclusion of both synovial fluid and peripheral blood immune responses to characterise local and systemic inflammatory activity.
- The matched-subject design using the contralateral pain-free knee as an internal control reduces inter-individual biological variability and strengthens causal inference regarding pain-related immune responses.
- Standardised sample handling, stimulation protocols and centralised laboratory analyses enhance reproducibility, while blinding of laboratory investigators to clinical status minimises measurement bias.
- Strict eligibility criteria and exclusion of comorbid conditions that affect immune activity improve internal validity but may limit generalisability to more complex, multimorbid arthroplasty populations.
- Absence of radiographic confirmation of osteoarthritis in asymptomatic controls may allow subclinical disease, potentially biasing results towards the null and underestimating true pain-biomarker associations.

## Introduction

 Knee osteoarthritis is one of the most prevalent chronic degenerative joint disorders globally and represents a leading cause of pain, functional decline and disability.^1^ Total knee arthroplasty (TKA) is a well-established intervention, designed to relieve pain and restore mobility.^2^,^4^ However, postoperative outcomes remain variable and not universally satisfactory. Approximately 10–15% of patients with TKA report persistent pain or dissatisfaction following surgery, despite otherwise successful radiographic and functional results.^5^,^9^ A considerable contributor to this variability is the inherently subjective nature of pain assessment, which relies predominantly on patient-reported scores such as the Visual Analogue Scale and Numerical Rating Scale (NRS). While these measures are clinically practical, they are influenced by psychological state, personality traits and contextual factors, potentially obscuring the true biological driver of pain.^10^,^12^ Literature also suggests that preoperative patient-reported pain intensity prior to TKA is only moderately related to radiographic knee osteoarthritis severity^13^,^17^ and mostly related to female sex and psychological symptoms including anxiety, depression and pain catastrophising symptoms.^18 19^ This creates uncertainty in pain evaluation and decision-making regarding the timing and necessity of joint replacement surgery, and complicates the interpretation of recovery trajectories.

Growing evidence over the past two decades has revealed that pain perception is not exclusively a central nervous system phenomenon but arises from a bidirectional neuroimmune interface in which peripheral immune activity mirrors and modulates nociceptive processing in the brain.^20^ Animal and human studies have demonstrated the involvement of glial activation,^21^,^24^ Toll-like receptor (TLR) expression and peripheral cytokine release in chronic pain pathways.^25^,^27^ In individuals with dysmenorrhoea and other chronic pain states, stimulated immune responses, particularly interleukin-1β (IL-1β) release from peripheral blood mononuclear cells, were found to be substantially elevated compared with pain-free controls.^28 29^ These findings suggest that inflammatory signatures may serve as objective biomarkers of pain intensity. Despite this progress, no studies to date have explored whether similar immune responses occur in somatic pain conditions such as osteoarthritis-related knee pain. This represents a critical knowledge gap, given that osteoarthritis pain is clinically heterogeneous: some patients report severe pain despite minimal radiographic degeneration, whereas others with advanced joint damage experience surprisingly low symptom burden.^30 31^ Objective pain biomarkers could provide insight into this discrepancy, supporting more personalised surgical decision-making and postoperative care and pain management.

The primary aim of this exploratory study is to investigate whether peripheral immune responses, IL-1β expression quantified from both peripheral blood mononuclear cells (PBMCs) and synovial fluid mononuclear cells (SFMCs), differ between patients with symptomatic knee osteoarthritis awaiting TKA and pain-free healthy controls. We hypothesise that stimulated immune responses (IL-1β release) are elevated in patients with symptomatic knee osteoarthritis compared with pain-free controls. Secondary exploratory analyses will (1) examine associations between immune responses, subjective pain measures and psychological factors and (2) evaluate hyperspectral (HS) imaging as a complementary approach for characterising immune cell phenotypes.^32^ This study is intended as a hypothesis-generating study to inform future research on objective pain biomarkers in orthopaedic populations, and to address the absence of objective, biologically anchored pain measurement in orthopaedic surgery. This exploratory study applies neuroimmune biomarker technology to musculoskeletal pain, and may provide a foundation for integrating objective biological markers with subjective pain assessment to improve clinical decision-making and ultimately contribute to a more multidimensional approach of pain assessment.

## Methods and analysis

### Aim, design and setting of the study

This study concerns an exploratory cross-sectional matched-subjects observational study and will be performed in accordance with the Declaration of Helsinki guidelines, and reported in accordance with Strengthening the Reporting of Observational Studies in Epidemiology (STROBE) guidelines. All aspects of this study have been approved by the Southern Adelaide Clinical Human Research Ethics Committee (SAC HREC), Southern Adelaide Local Health Network (SALHN) (2024/HRE00253). Trial registration at the Australian and New Zealand Clinical Trials Registry was completed before commencement of participant recruitment and enrolment (Trial Registration Number: ACTRN12626000084381, date: 22–01-2026. Universal Trial Number: U1111-1332-7067).

Recruitment is planned to commence in March 2026 and is expected to continue for approximately 6 months. Laboratory analyses will be performed in batches during and after recruitment. The investigators performing the laboratory analyses are blinded to the clinical status of the patients. Participant enrolment, clinical assessments, and sample collection will be conducted in Noarlunga Hospital (Flinders Medical Centre), Adelaide. Investigators will not be blinded to group allocation (group with patients awaiting TKA or group with healthy controls). Sample analyses and data analyses will be performed in the Neuroimmunopharmacology Laboratory of Helen Mayo South Building, University of Adelaide.

The primary aim of this exploratory study is to assess whether peripheral immune response parameters (IL-1β release following TLR-2 and TLR-4 activation) differ between painful osteoarthritic knees and pain-free controls. This will be conducted through measuring peripheral immune responses (pain biomarkers, ie, IL-1β expression), quantified from both PBMCs and SFMCs following TLR-2 and TLR-4 activation using PAM3CSK4 (PAM3, a synthetic lipopeptide which functions as an agonist for TLR-2) and lipopolysaccharide (LPS, ie, endotoxin, gram-negative bacterial cell wall component which functions as an agonist for TLR-4), between patients with symptomatic knee osteoarthritis awaiting TKA and pain-free healthy controls.

Secondary exploratory aims include (1) assessing associations between immune responses (IL-1β expression) and subjective pain scores, (2) exploring relationships between immune responses and psychological factors (anxiety, depression, pain catastrophising) and (3) evaluating HS imaging as a complementary approach for characterising immune cell phenotypes by assessing the relationship between HS imaging hues and IL-1β expression.

### Patient and public involvement

Patients and the public were not involved in the design of this study, including the development of the research question, outcome measures, study design or recruitment strategy. The study was designed based on identified gaps in objective pain assessment in osteoarthritis and feasibility within standard clinical care pathways. Participants are not scheduled to be involved in the conduct of the study. Results will be disseminated via peer-reviewed publication and scientific presentations. Where feasible, a summary of the study findings will be made available to participants on request.

### Participants and eligibility criteria

Participants will be consecutively included after screening for eligibility and provided informed consent at the orthopaedic clinic of Noarlunga Hospital, Flinders Medical Centre, SALHN. The study planned to enrol 3 groups of participants: (1) 20 patients awaiting TKA including 20 *painful* knees with moderate to severe osteoarthritis, (2) 20 controls, the healthy contralateral *pain-free* knee of these 20 patients awaiting TKA and (3) 20 different healthy controls for venous blood measurements, age and sex matched healthy individuals with no pain complaints in general at that moment, and no function limitation or pain in any joint (no diagnosed osteoarthritis in both knees). This will result in 40 participants in total: Group 1: 20 patients with 20 *painful* knees with osteoarthritis that are awaiting TKA and 20 *pain-free* contralateral control knees of the same patients; Group 2: 20 *pain-free* control patients without any knee complaints.

Healthy controls are defined as individuals without knee pain or functional limitations in either knee. Radiographic imaging will not be performed in this group, as the primary focus of the study is the biological correlates of pain rather than structural osteoarthritis, and obtaining radiographs in asymptomatic individuals solely for research purposes would expose participants to unnecessary radiation. It is acknowledged that some asymptomatic individuals may have radiographic osteoarthritis; however, structural changes and pain symptoms are known to correlate only moderately.^13^,^17^

Inclusion criteria for eligibility for this study are (1) awaiting a unilateral primary TKA for symptomatic (painful) advanced osteoarthritis,^33^ (2) asymptomatic (pain-free) contralateral knee without functional limitation or pain (no or mild osteoarthritis), (3) otherwise good general health, (4) aged 18 years or over, (5) completion of all preoperative questionnaires and (6) providing informed consent.

Exclusion criteria for all subjects include (1) TKA indication other than symptomatic osteoarthritis for painful knee, (2) painful osteoarthritis in any other joint, (3) other relevant pain complaints elsewhere, (4) presence of any auto-immune diseases, for example, rheumatoid arthritis, lupus, inflammatory bowel disease, psoriasis, etc, (5) one or more of the following comorbidities: diabetes, gout or septic or reactive arthritis in any joint or any clinically significant renal, hepatic, cardiac disease, (6) a medical history including: fracture or surgery to either of the knees, recent (<3 months) major surgery or significant trauma, (7) presence of an inflammatory process, or clinically significant infection in the last 4 weeks, (8) immunisation within the last 4 weeks, (9) current use of immunosuppressant medication such as a steroid, hydroxychloroquine, methotrexate or azathioprine, (10) inability to read or comprehend the written information provided, (11) current use of medications known to affect innate immune responsiveness including amitriptyline, chemotherapy or Tumor Necrosis Factor Alpha (TNF-α) inhibitors or (12) current pregnancy.

### Recruitment and enrolment procedures

Initial recruitment of 20 patients awaiting a unilateral primary TKA for symptomatic (painful) advanced osteoarthritis at Noarlunga Hospital—SALHN will be via knee arthroplasty surgeons at the orthopaedic department. The recruitment of the 20 healthy controls without knee symptoms in both knees will also be performed via knee arthroplasty surgeons at the orthopaedic department of Flinders Medical Centre and Noarlunga Hospital.

All of these patients will be approached by email, which will include links to the study information. An additional email will be sent to all interested patients willing to participate, including the informed consent form, and a more detailed overview of the study.

For all participants who complete informed consent, eligibility will be reviewed through the electronic health-record system according to the inclusion and exclusion criteria, and baseline patient demographics (age, gender, body mass index [BMI], smoking, American Society of Anesthesiologists (ASA) physiological status classification, side of painful and pain-free control knee and other comorbidities) and preoperative medication, specifically pain medication, will be collected. Subsequently all patients (n=40) will be contacted by phone to confirm the presence and absence of all inclusion criteria and exclusion criteria, respectively.

### Clinical assessment and questionnaires

The primary outcome included EC50 (concentration-response parameter: concentration which produces 50% of the maximal effect) for IL-1β release, to determine the responsiveness of PBMC and SFMC to LPS and PAM3 stimulation. Osteoarthritis severity of the painful knee will be graded by an orthopaedic surgeon based on Kellgren-Lawrence and self-assessed pain scores will be determined by the patient preoperatively for both knees using the NRS-pain scores. Additionally, preoperative mental state will be assessed by completing two Patient Reported Outcome Measures (PROM): the Hospital Anxiety and Depression Scale (HADS) questionnaire,^34^ and the Pain Catastrophizing Scale (PCS) questionnaire.^35 36^ The HADS consists of an anxiety (HADS-A) and a depression (HADS-D) subscale, each including seven items scoring 0–3, resulting in a total maximum score of 21 per subscale. Scores of 8–11 are considered borderline anxiety or depression, whereas scores>11 are considered presumable anxiety or depression.^37^ In this study, an HADS-A or HADS-D score ≥8 will be categorised as the presence of anxiety or depression, respectively.^34 38^ The PCS is a validated questionnaire consisting of 13 statements regarding feelings and thoughts during experiencing pain.^35 36^ Patients rate these statements on a 5-point Likert scale ranging from ‘not at all’ to ‘all the time’ with the total score ranging from 0 (no catastrophising) to 52 (severe catastrophising). A score of 30 or higher is considered clinically relevant catastrophising;^36 39^ as such, patients will be considered pain catastrophizing (PC) or non-PC patients.

### Sample collection

Preoperatively, at the pre-admission clinic 2–4 weeks before surgery, enrolled TKA-awaiting patients (n=20) will receive routine venous blood sampling for elective surgery preparations at Noarlunga Hospital. At that same blood collection moment, a total of 30 mL of blood will be collected for this study: 10 mL for the functional immune stimulation assay used to quantify cytokine release (IL-1β) (ie, ‘painCELL test’), 10 mL for the HS imaging-based analysis of immune cells used to characterise cellular autofluorescence signatures and identify potential phenotypic differences between participant groups (ie, ‘painHS test’) and 10 mL for storage and backup in case of experimental assay complications. Also, all preoperative questionnaires (HADS, PCS and NRS-pain) will be completed during this visit.

Subsequently, perioperatively during the elective TKA surgery, synovial fluid will be collected from the painful knee receiving TKA surgery, just after opening the knee joint. A total of 30 mL of synovial fluid will be collected for this study: 10 mL for the painCELL test, 10 mL for the painHS test and 10 mL for storage and backup in case of experimental assay complications.

Similarly, the contralateral pain-free knee will be punctured for 5–30 mL synovial fluid directly after surgery when the patient is still under narcosis, so that the patient will experience no additional discomfort. This procedure is included to enable within-subject comparison between painful osteoarthritic and pain-free joints within the same patient, thereby reducing inter-individual variability in immune response measurements. The aspiration will be performed under sterile operating theatre conditions by an experienced orthopaedic surgeon, following standard aseptic procedures to minimise the risk of complications such as infection.

All healthy blood-control individuals without pain symptoms in both knees (n=20) will attend Flinders Medical Centre or Noarlunga Hospital, where similarly venous blood samples will be collected for the study, which includes 30 mL of blood in total: 10 mL for the painCELL test, 10 mL for the painHS test and 10 mL for storage and backup in case of experimental assay complications.

### Laboratory procedures

#### PainCELL sample analysis

Venous blood collected in EDTA-coated vacutainer tubes and the synovial fluid collected in sodium citrate-coated tubes/sterile containers for the painCELL test will immediately be inverted several times to prevent blood from clotting. For the PBMC and SFMC isolation success to be maximised, the blood and synovial fluid will be cooled to room temperature before the cell isolation procedures initiate (30–60 min, depending on the ambient temperature). PBMCs are isolated using Optiprep density medium (Sigma-Aldrich #D1556; Castle Hill, NSW, Australia) as directed by the manufacturer using the mixer flotation method, as previously described.^23 26 27^ Due to the expected considerably small amount of synovial fluid available from sampling, this last step using Optiprep will not be performed for the SFMCs.

Isolated PBMCs and all SFMCs are diluted to 10^6^ cells/mL in enriched Roswell Park Memorial Institute Medium (RPMI) 1640 (10% fetal bovine serum, 2 mM l-glutamine and 1% penicillin/streptomycin) and plated into 96 well plates (100 µL per well). Various concentrations of innate immune stimulants will be added into the wells which include a TLR-2 agonist (Pam3; Merck #506350; Darmstadt, Germany; from 12.5 pg/mL to 1 mg/mL), and a TLR-4 agonist (LPS; InvivoGen #TLRL-3PELPS; San Diego, California, USA; from 12.5 pg/mL to 10 mg/mL) in a final volume of 200 mL/well. Plates are then incubated for 24 hours, following which 150 mL of the supernatant will be removed, transferred to a separate 96 well plate and stored at −80°C for later analysis. Supernatants are analysed using a Human Interleukin 1 eta (IL-1β) OptEIA ELISA Set II (BD Biosciences #557953; San Jose, California, USA) per the manufacturer’s instructions. The standard curve range for this assay is estimated to be 3.9–500 pg/mL. Supernatants are added to the ELISA either neat or diluted 1:10 to ensure the quantified values fall within the limits of the standard curve.

#### PainHS sample analysis

Venous blood samples collected in EDTA-coated vacutainer tubes and the synovial fluid collected in sodium citrate-coated tubes/sterile containers for the painHS test were frozen and stored at −80°C for later analysis. On the day of analysis, samples will be thawed in a 37°C water bath for 45 mins. The blood and synovial fluid will be washed with equal volumes of RPMI1640, centrifuged (240×g for 5 min) with supernatant removed and resuspended in 3–5 mL of RPMI. Cells of the blood and synovial fluid samples are counted and then placed on the imaging slides for HS imaging. An adapted fluorescence microscope (Olympus iX71) will be used with a 40×water U12 series objective (with wide transmission in UV range). LEDs with excitation wavelength bands centred at 334, 365, 385, 395, 405, 415, 425, 435, 455, 475, 495 nm, each about 10 nm wide produce close to monochromatic light to excite the sample’s auto-fluorescence. The corresponding sample emission will be measured with three epifluorescence filter cubes with the emission bands centred at 447 nm (60 nm wide), 587 nm (35 nm wide) and 700 nm long pass. Overall, in this work we use 18 spectral channels (ref) with the same channel numbering as used here,^40^ and each field of view will be imaged in all these 18 channels forming a HS image. Images are captured by a digital camera C1140, OCRA Flash 4.0 (Hamamatsu). Images will then be saved and digitally transferred for digital analysis by the Neuroimmunopharmacology laboratory.

### Sample size calculation

A sample size calculation based on achieving 90% power and a Type 1 error rate of 5% was chosen. The calculation was based on primary analyses and determined that 24 participants in total (12 participants per group) would be required to detect a 2.40 difference in the Least Squares means for EC50 (concentration-response parameter: concentration which produces 50% of the maximal effect, to determine the responsiveness of PBMC and SFMC to LPS and PAM3 stimulation) assuming a within-group SD of 1.78. The clinically significant difference in EC50 was estimated based on parameters described by Evans *et al*.^29^ To account for a potential 10% loss to follow-up and safety margin with regards to sample analyses, a total sample of 40 participants (20 per group) is to be recruited.

It should be noted that the calculated sample size was based on the primary analysis and may not provide sufficient statistical power for all planned secondary analyses, such as investigating associations between immune responsiveness (PBMCs and SFMCs responses) and psychological symptoms including anxiety, depression or pain catastrophising. General statistical guidance for multivariable modelling recommends an adequate number of observations per covariate to ensure model stability (eg, 10 patients per use of 1 covariate). Therefore, the number of covariates included in secondary analyses will be kept limited (only one psychological variable per model) to maintain analytical feasibility. As these secondary analyses are exploratory in nature, their findings will be interpreted accordingly. The present study represents an initial exploratory investigation of neuroimmune pain biomarkers within an orthopaedic population.

### Data analysis plan

Primary analyses aim to assess whether peripheral immune response parameters differ between painful osteoarthritic knees and pain-free controls. This will be evaluated using both within-subject comparisons (painful knee vs contralateral pain-free knee in patients undergoing TKA for synovial fluid analyses) and between-group comparisons (patients vs healthy controls for peripheral blood analyses). Secondary analyses will explore associations between immune response parameters and PROMs for pain intensity and psychological factors including anxiety, depression and pain catastrophising. All secondary analyses, including multivariable and machine-learning approaches, are exploratory in nature and aimed at hypothesis generation rather than confirmatory inference.

#### Primary outcome analysis: painCELL data

Concentration-response curves from the in vitro innate immune stimulated IL-1β release from PBMCs and SFMCs are calculated using GraphPad Prism version 8 (MathWorks; Natick, Massachusetts, USA). Differences between pain-free controls and painful symptomatic osteoarthritis patients (within the same patient for synovial fluid samples and a different patient for blood samples) will be assessed on EC_50_, Hillslope and ‘Top’ (or E_max_). The ‘Bottom’ value will be constrained to a constant of 0. An F-test in GraphPad Prism will be performed to determine if these values should be the same or different between the participant groups. The goodness of fit obtained from the TLR-2 stimulation is not sufficient to warrant presentation in the results, owing to lower-than-expected cytokine expression in the assays.

The painCELL assay cytokine data will be combined with osteoarthritis severity and NRS-pain reports, as well as other demographics (such as mental state) for each participant in a data file. Additional parameterisation of the data set will be performed to capture the delta change in cytokine release between low and high concentration innate immune challenges and between these delta values and the steepness of the concentration response.

First, a feature selection (dimensionality reduction) step will be performed to remove highly correlated variables, by examining the correlation matrix and applying the ‘Bivariate Correlations’ function to identify and exclude variables with Pearson Correlation coefficients greater than 0.8. For each specific hypothesis test, data subsets will be created using the ‘Select Cases’ function to exclude irrelevant subject data (eg, when testing the model for Healthy synovial sample Controls and the contralateral painful knee, Healthy blood sample Controls data will be excluded).

For analysis, a randomised approach with at least a 20% holdout validation and test dataset will be employed. The dataset will be split to ensure that the test dataset is not included in the model training phase. For the categorical selection hypotheses, several classification techniques will be applied in parallel, expected to include: Linear Discriminant Analysis (LDA), Classification and Regression Trees, K-Nearest Neighbours, Support Vector Machine and Random Forest models. The best-performing model will be identified based on the highest accuracy and lowest error rate, for which a confusion matrix will then be created.

#### Secondary outcome analysis: painHS data

HS images are pre-processed as previously described.^41^ This will be followed by cell segmentation, carried out in an automatic approach using a parametric watershed algorithm with a partial set principal components (PCA) image. The channels employed in the PCA segmentation image are found through trial and test. This segmentation protocol will be augmented with additional manual segmentation using the phase contrast image and selecting cells at random. By estimation, this will yield 70–90 cells from each image, belonging to one of the following four sample groups: (1) blood samples from painful symptomatic osteoarthritis patients (participant group 1), (2) synovial fluid samples from painful symptomatic osteoarthritis patients (participant group 1), (3) synovial fluid samples from contralateral pain-free control knee (participant group 1) and (4) blood samples from pain-free controls (participant group 2).

After each cell is segmented from the background, a number of quantitative features are calculated using the segmented cells to parameterise the cell data, with the primary goal of delineating participant population membership. In particular, for each cell we will calculate ratios of mean channel intensities of pairs, correlation of channel pixel intensities and coherence of the statistics of the channel pairs. The feature data for all cells will then undergo a singular value decomposition as described previously.^40^ These uncorrelated variables are then further used for supervised discriminatory analysis.^40 41^ The LDA will provide another set of seven feature variables maximising the separation between the sample populations.^40^ These are then optimally projected by using the second linear discriminant analysis onto two dimensions to obtain a graphical representation, showing the optimal participant group separation. Subsequently, they are projected again using LDA on a single optimised dimension. Receiver operator curves will be plotted to assess the sensitivity and specificity of the approach.

## Ethics and dissemination

Ethical approval for this study was granted by the Southern Adelaide Clinical Human Research Ethics Committee (SAC HREC), Southern Adelaide Local Health Network (SALHN), Australia, under approval number 2024/HRE00253 (approval date 13 June 2025).

Site-level governance authorisation was subsequently obtained via Site Specific Assessment under approval number 2024/SSA00641, authorising project commencement 05 December 2025, for a period of 3 years.

All participants will provide written informed consent prior to inclusion.

No participant data have yet been generated, as participant recruitment and sample collection have not yet commenced. On completion of the study, de-identified datasets containing clinical, questionnaire and biomarker data (including peripheral immune and HS pain-signal measures analysed at the Neuroimmunopharmacology Laboratory, University of Adelaide) will be created. Because individual-level pain biomarker data may carry potential identifiability risk, the full dataset will not be made publicly available. However, de-identified data supporting the findings of the final study will be made available from the corresponding author on reasonable request, subject to approval by the SALHN governance office and compliance with ethical conditions and data-sharing regulation. Aggregated summary results will be included in the final publication, and all analytic code and statistical methods can be shared on request.

The study findings will be disseminated through publication in peer-reviewed scientific journals and presentations at national and international research conferences. Where appropriate, a plain-language summary of the study results will be made available to interested participants on request.

## Data Availability

Data sharing not applicable as no datasets generated and/or analysed for this study.
