# Supplementary material for: Protocol for assessment of pain biomarkers in orthopaedic patients undergoing total knee arthroplasty in a tertiary care hospital and neuroimmunopharmacology laboratory in South Australia: a cross-sectional matched-subject observational study
Source: BMJ Open. 2026 Jun 22;16(6):e117858. doi: 10.1136/bmjopen-2026-117858 (PMC13288962; doi:10.1136/bmjopen-2026-117858)
Supplement: Supplementary file 1 [file bmjopen-2026-117858.reviewer_comments.pdf]

PEER REVIEW HISTORY

BMJ Open publishes all reviews undertaken for accepted manuscripts. Reviewers are asked to complete a checklist review form and are provided with free text boxes to elaborate on their assessment. These free text comments are reproduced below.

ARTICLE DETAILS

Title (Provisional)

Protocol for Assessment of Pain Biomarkers in Orthopaedic Patients Undergoing Total Knee Arthroplasty in a Tertiary Care Hospital and Neuroimmunopharmacology Laboratory in South Australia: A Cross-sectional Matched-subject Observational Study

Authors

Aalders, Margot B; Van Marle, Laura; Evans, Samuel G; Wilson, Christopher; Lin, Indy; Verjans, Johan W.; Doornberg, Job N; Hutchinson, Mark; Jaarsma, Ruurd L.

VERSION 1 - REVIEW

**Reviewer**	**1**
**Name**	**Qian, Wenwei**
**Affiliation**	**Peking Union Medical College Hospital**
**Date**	**06-Mar-2026**
**COI**	**None**

Thanks author for allowing me to review this interesting research proposal. This study will investigate whether peripheral immune responses can serve as objective pain biomarkers in patients with painful knee osteoarthritis compared with pain-free controls, and how these markers relate to anxiety, depression and pain catastrophising. The only concern is regarding the sample size. It is still unclear whether the limited sample size can have sufficient test power.

**Reviewer**	**2**
**Name**	**Ramadanov, Nikolai**
**Affiliation**	**University Hospital Brandenburg/Havel Brandenburg Medical School**
**Date**	**10-Mar-2026**
**COI**	**None**

This study protocol investigates whether peripheral immune responses may serve as objective biomarkers of pain in patients undergoing total knee arthroplasty (TKA). The topic is relevant and the integration of immunological assays, psychological PROMs, and hyperspectral imaging represents an innovative approach. The protocol is generally well structured and prospectively registered. However, several aspects require clarification.

1. The study is described as a cross-sectional matched-subject observational study. The manuscript should clarify whether the primary analysis focuses on within-subject comparisons (painful vs contralateral knee) or between-group comparisons (patients vs healthy controls).

2. The planned sample size (n=40 participants) appears relatively small considering the complexity of the proposed analyses, including multivariable modelling and machine-learning approaches. Additional justification would be helpful.

3. The machine-learning pipeline is described in considerable detail. For a protocol paper, a simplified description of the planned analytical strategy may improve clarity.

4. The protocol includes aspiration of synovial fluid from the contralateral pain-free knee during surgery. You should briefly clarify the ethical justification and risk mitigation for this procedure.

5. Healthy controls are defined as individuals without knee pain. It should be clarified whether radiographic osteoarthritis will be excluded in these participants.

6. The introduction focuses mainly on neuroimmune mechanisms of pain. Additional contextualisation within the orthopaedic literature on factors influencing TKA outcomes may strengthen the manuscript.

7. Recent orthopaedic studies investigating biomechanical factors during TKA could be considered. For example, Becker et al. quantified distraction forces during ligament-balanced TKA and reported reference values of approximately 150 N in extension and 140 N in flexion, highlighting the importance of intraoperative soft-tissue management.

8. Minor language revisions are recommended, and project-specific terms such as "painCELL" and "painHS" may benefit from brief clarification.

VERSION 1 - AUTHOR RESPONSE

Reviewer: 1

Dr. Wenwei Qian, Peking Union Medical College Hospital

Comments to the Author:

Thanks author for allowing me to review this interesting research proposal. This study will investigate whether peripheral immune responses can serve as objective pain biomarkers in patients with painful knee osteoarthritis compared with pain-free controls, and how these

markers relate to anxiety, depression and pain catastrophising. The only concern is regarding the sample size. It is still unclear whether the limited sample size can have sufficient test power.

Response to reviewer 1:

We thank the reviewer for the positive assessment of our study protocol and for raising the question regarding the adequacy of the planned sample size. We performed a formal sample size calculation, which is described in the Methods section of the manuscript under "Sample size calculation". In line with standard practice for sample size analyses, the calculation was based on primary analyses detecting differences in the primary outcome parameter EC50, which reflects the responsiveness of peripheral blood mononuclear cells (PBMCs) and synovial fluid mononuclear cells (SFMCs) to innate immune stimulation (TLR-2 and TLR-4). Based on parameters reported by Evans et al. (also cited in the manuscript), a clinically meaningful difference of 2.40 in least squares means for EC50 was assumed with a within-group standard deviation of 1.781. Using a significance level of $\alpha=0.05$ and 90% statistical power, the required sample size was 24 participants (12 per group).

However, to provide an additional safety margin and account for potential sample loss or technical variability in the laboratory assays, the planned recruitment was increased to 40 participants (20 per group). Therefore, the proposed sample size exceeds the number required by the a priori power calculation for the primary outcome analysis, and we are convinced that this sample size will have sufficient test power to explore the difference in responsiveness of PBMCs and SFMCs.

To improve clarity for readers, we have slightly expanded the explanation of the sample size rationale in the revised manuscript.

Added to line 329:

"(…) was based on primary analyses and (…)"

Added to line 337-345:

"It should be noted that the calculated sample size was based on the primary analysis and may not provide sufficient statistical power for all planned secondary analyses, such as investigating associations between immune responsiveness (PBMCs and SFMCs responses) and psychological symptoms including anxiety, depression, or pain catastrophizing. General statistical guidance for multivariable modelling recommends an adequate number of observations per covariate to ensure model stability (e.g., 10 patients per use of 1 covariate). Therefore, the number of covariates included in secondary analyses will be kept limited (only

one psychological variable per model) to maintain analytical feasibility. As these secondary analyses are exploratory in nature, their findings will be interpreted accordingly. The present study represents an initial exploratory investigation of neuroimmune pain biomarkers within an orthopaedic population.”

References

1. Evans SF, Kwok YH, Solterbeck A, Liu J, Hutchinson MR, Hull ML, Rolan PE (2020) Toll-Like Receptor Responsiveness of Peripheral Blood Mononuclear Cells in Young Women with Dysmenorrhea. J Pain Res 13:503–516

Reviewer: 2

Dr. Nikolai Ramadanov, University Hospital Brandenburg/Havel Brandenburg Medical School, Brandenburg Medical School Theodor Fontane

Comments to the Author:

This study protocol investigates whether peripheral immune responses may serve as objective biomarkers of pain in patients undergoing total knee arthroplasty (TKA). The topic is relevant and the integration of immunological assays, psychological PROMs, and hyperspectral imaging represents an innovative approach. The protocol is generally well structured and prospectively registered. However, several aspects require clarification.

Comment 1: The study is described as a cross-sectional matched-subject observational study. The manuscript should clarify whether the primary analysis focuses on within-subject comparisons (painful vs contralateral knee) or between-group comparisons (patients vs healthy controls).

Response 1: We thank the reviewer for the positive assessment of our study protocol and for this valuable remark. The primary objective of this study is to evaluate whether peripheral immune responses differ between painful osteoarthritic knees and pain free knees. Accordingly, the primary analyses will compare immune response parameters between painful osteoarthritic knees and pain-free controls. This includes both within-subject comparisons (painful knee versus contralateral pain free knee within the same patient for synovial fluid samples) and between-group comparisons (patients versus healthy controls for peripheral blood samples).

We have clarified this in the revised manuscript by explicitly describing the primary analytical comparisons under “Data analysis plan”, line 347-353:

“Primary analyses aim to assess whether peripheral immune response parameters differ between painful osteoarthritic knees and pain-free controls. This will be evaluated using both within-subject comparisons (painful knee versus contralateral pain-free knee in patients undergoing TKA for synovial fluid analyses) and between-group comparisons (patients versus healthy controls for peripheral blood analyses). Secondary analyses will explore associations between immune response parameters and PROMs for pain intensity and psychological factors including anxiety, depression and pain catastrophising.”

Comment 2: The planned sample size (n=40 participants) appears relatively small considering the complexity of the proposed analyses, including multivariable modelling and machine-learning approaches. Additional justification would be helpful.

Response 2:

We appreciate the reviewer’s detailed assessment of the manuscript and for raising the question regarding the adequacy of the planned sample size. We performed a formal sample size calculation, which is described in the Methods section of the manuscript under “Sample size calculation”. In line with standard practice, the calculation was based on the primary analysis, which assesses differences in the primary outcome parameter EC50, reflecting the responsiveness of peripheral blood mononuclear cells (PBMCs) and synovial fluid mononuclear cells (SFMCs) to innate immune stimulation (TLR-2 and TLR-4). Based on parameters reported by Evans et al. (also cited in the manuscript), a clinically meaningful difference of 2.40 in least squares means for EC50 was assumed with a within-group standard deviation of 1.78 [1]. Using a significance level of $\alpha=0.05$ and 90% statistical power, the required sample size was 24 participants (12 per group). To provide an additional safety margin and account for potential sample loss or technical variability in the laboratory assays, the planned recruitment was increased to 40 participants (20 per group). Therefore, the proposed sample size exceeds the number required by the a priori power calculation for the primary outcome analysis, and we are confident that this sample size provides sufficient statistical power to detect biologically meaningful differences in PBMCs and SFMCs.

The additional secondary analyses, including multivariable and machine-learning approaches, are intended as exploratory analyses aimed at identifying potential multidimensional patterns between immune markers, clinical variables, and psychological factors. These analyses are hypothesis-generating and do not determine the required sample size. As such, their findings will be interpreted with appropriate caution. We

acknowledge that traditional statistical guidelines for multivariable modelling often recommend a sufficient number of observations per covariate to ensure model stability. In this study, the multivariable analyses will therefore remain limited in scope and will include only a small number of predefined covariates (including only one psychological variable per model) to maintain analytical feasibility. Given that this study represents an initial exploratory investigation of neuroimmune pain biomarkers in an orthopaedic population, we consider this approach appropriate.

To address the reviewer's concern and clarity for readers, we have expanded the explanation of the sample size rationale in the revised manuscript and clarified that the multivariable and machine-learning analyses are exploratory.

Added to line 329:

"(…) was based on primary analyses and (…)"

Added to line 337-345:

"It should be noted that the calculated sample size was based on the primary analysis and may not provide sufficient statistical power for all planned secondary analyses, such as investigating associations between immune responsiveness (PBMCs and SFMCs responses) and psychological symptoms including anxiety, depression, or pain catastrophizing. General statistical guidance for multivariable modelling recommends an adequate number of observations per covariate to ensure model stability (e.g., 10 patients per use of 1 covariate). Therefore, the number of covariates included in secondary analyses will be kept limited (only one psychological variable per model) to maintain analytical feasibility. As these secondary analyses are exploratory in nature, their findings will be interpreted accordingly. The present study represents an initial exploratory investigation of neuroimmune pain biomarkers within an orthopaedic population."

Comment 3: The machine-learning pipeline is described in considerable detail. For a protocol paper, a simplified description of the planned analytical strategy may improve clarity.

Response 3:

We thank the reviewer for this helpful suggestion. We agree that the previous description of the machine-learning analysis contained a level of technical detail that may not be necessary in a study protocol manuscript. To improve clarity and readability, we have simplified the description of the analytical strategy in the revised manuscript. The revised text now

provides a concise overview of the planned analytical approach, focusing on the overall strategy of exploratory pattern identification and classification of immune-response profiles, while omitting some of the more detailed technical steps. The underlying analytical framework and planned modelling approaches remain unchanged.

Line 381, deleted: "(…) using the 'Split File' function, ensuring (…)"

Line 384, deleted: "(…) using the 'Discriminant Analysis' function (…)"

Line 384, deleted: "(…) using the 'Decision Trees' function (…)"

Line 384, deleted: "(…) using the 'K Nearest Neighbors' function (…)"

Line 385, deleted: "(…) using the 'SVM' extension available through SPSS Modeler (…)"

Line 385, deleted: "(…) using the 'Random Trees' extension also available through SPSS Modeler (…)"

Line 386, added: "(…), for which a"

Line 386, deleted: "(…). A"

Line 387, deleted: "(…) for the best model by applying it to the separate validation dataset using the 'Crosstabs' function. Full outputs of the best-fit model, including classification tables and accuracy measures, will be provided in the Appendix of the main manuscript"

Additionally, for optimization purposes, we edited the following sentences as well:

Line 400, added "(…) the following (…)" and added "(…) sample (…)". Deleted "participant".

Line 401: added "(participant group 1)"

Line 402: added "(participant group 1)". Added "synovial fluid samples from". Deleted "group 2".

Line 403: added "(participant group 1)". Deleted "for synovial fluid samples".

Line 404: deleted "of different patients", and added "(participant group 2'.

Line 428: deleted "(…) / PCA" and ", where the new variables satisfied the variance maximisation criteria and are used in creating the unsupervised plots".

Comment 4: The protocol includes aspiration of synovial fluid from the contralateral pain-free knee during surgery. You should briefly clarify the ethical justification and risk mitigation for this procedure.

Response 4: We thank the reviewer for this important comment. The aspiration of synovial fluid from the contralateral pain-free knee was included to allow within-subject comparison of immune responses between painful osteoarthritic and pain-free joints within the same patient. This design reduces inter-individual biological variability and increases statistical efficiency for the primary analyses.

To minimise risk to participants, the aspiration will be performed while the patient is already under general anaesthesia during the surgical procedure and under sterile operating theatre conditions, by an experienced orthopaedic surgeon. The procedure follows the same aseptic principles as routine intra-articular procedures, and the risk of complications such as infection is considered very low. These aspects were reviewed and approved by the institutional Human Research Ethics Committee (SALHN HREC).

To clarify this point, we have added a brief explanation of the ethical justification and risk mitigation strategy in the Methods section of the revised manuscript, under “Sample collection”.

Line 275-281, added: “(…) experience no additional discomfort. This procedure is included to enable within-subject comparison between painful osteoarthritic and pain-free joints within the same patient, thereby reducing inter-individual variability in immune response measurements. The aspiration will be performed under sterile operating theatre conditions by an experienced orthopaedic surgeon, following standard aseptic procedures to minimise the risk of complications such as infection.”. Deleted: “(…) suffer as little as possible”.

And additionally, for optimization purposes, we edited the following sentence:

Line 273, change “punctuated” to “punctured”, change “30” to “5-30”.

Response 5: We thank the reviewer for this thoughtful comment. In the present study, healthy controls are defined based on the absence of knee pain or functional limitations rather than radiographic findings. The primary objective of this study is to investigate immune responses associated with pain, rather than structural osteoarthritis itself. Therefore, group allocation is based on the presence or absence of clinically relevant pain symptoms. As such, radiographic imaging will not be performed in healthy control participants. This decision was made for ethical reasons, as obtaining knee radiographs solely for research purposes in asymptomatic individuals would expose participants to unnecessary radiation without clinical benefit. We acknowledge that some asymptomatic individuals may have radiographic osteoarthritis despite the absence of pain. However,

current evidence suggests that the association between structural osteoarthritis and pain symptoms is only modest [2-4] or even absent [5, 6], and radiographic findings alone do not necessarily reflect the presence of clinically relevant pain. Given that the primary research question focuses on biological correlates of pain rather than structural disease, defining controls based on the absence of knee pain was considered appropriate for this exploratory study.

To clarify this point for readers, we have added a short explanation to the Methods section of the revised manuscript, under "Participants and eligibility criteria":

Line 204, added: "Healthy controls are defined as individuals without knee pain or functional limitations in either knee. Radiographic imaging will not be performed in this group, as the primary focus of the study is the biological correlates of pain rather than structural osteoarthritis, and obtaining radiographs in asymptomatic individuals solely for research purposes would expose participants to unnecessary radiation. It is acknowledged that some asymptomatic individuals may have radiographic osteoarthritis; however, structural changes and pain symptoms are known to correlate only moderately [7, 9, 18, 19, 33]."

Additionally, optimized the methods section under 'Participants and eligibility criteria':

Line 197: deleted "(…), and 60 knees". Added "Group 1)" and "patients with 20" and "are".

Line 198: added "and". Deleted ",". Added "(…) Group 2)".

Line 199: deleted "without any knee complaints".

Comment 6: The introduction focuses mainly on neuroimmune mechanisms of pain. Additional contextualisation within the orthopaedic literature on factors influencing TKA outcomes may strengthen the manuscript.

Response 6: We thank the reviewer for this helpful suggestion. However, the primary objective of this study is to investigate the biological correlates of preoperative knee pain in patients with osteoarthritis rather than postoperative outcomes following total knee arthroplasty (TKA). Therefore, the introduction was intentionally focused on neuroimmune mechanisms underlying pain perception. Nevertheless, we do agree that the current phrasing may have created some unclarity regarding this matter, and additional contextualisation of pain intensity preoperatively in patients undergoing TKA may help readers better understand the clinical background of this study. In particular, several orthopaedic studies have demonstrated that the level of preoperative knee pain reported by

patients is only correlated modestly with the severity of radiographic osteoarthritis [2-6]. Instead, psychological factors such as anxiety, depression and pain catastrophizing, as well as demographic characteristics such as female sex, have been reported to influence the experience intensity of knee pain [7, 8].

To address the reviewer's suggestion while maintaining the focus of the manuscript, we have added some sentences to the Introduction describing current orthopaedic evidence regarding factors associated with preoperative pain in knee osteoarthritis.

Line 104-107, added "Literature also suggests that preoperative patient-reported pain intensity prior to TKA is only moderately related to radiographic knee osteoarthritis severity [7, 9, 18, 19, 33], and mostly related to female sex and psychological symptoms including anxiety, depression, and pain catastrophizing symptoms [1, 25]."

Line 107, deleted "Similarly, multiple studies in TKA populations showed a negative effect of psychological factors on postoperative pain, function or quality of life [1, 25]."

Line 107, added "(…) pain evaluation and (…)"

Line 109, deleted "postoperative".

Comment 7: Recent orthopaedic studies investigating biomechanical factors during TKA could be considered. For example, Becker et al. quantified distraction forces during ligament-balanced TKA and reported reference values of approximately 150 N in extension and 140 N in flexion, highlighting the importance of intraoperative soft-tissue management.

Response 7: We thank the reviewer for this interesting suggestion and for highlighting recent orthopaedic work investigating biomechanical factors during total knee arthroplasty. Studies examining intraoperative parameters such as ligament balancing and distraction forces are indeed very important for understanding surgical technique and postoperative joint mechanics. However, the primary objective of the present study is to investigate preoperative biological correlates of knee pain, specifically the relationship between peripheral immune responses, subjective pain measurements, and psychological factors in patients with knee osteoarthritis prior to TKA. As such, the focus of the manuscript is on mechanisms contributing to pain perception before surgery, rather than on intraoperative surgical factors or biomechanical aspects of the arthroplasty procedure itself. For this reason, we believe that detailed discussion of intraoperative biomechanical studies would fall outside the scope of the current protocol and could potentially distract from the primary

research question. We therefore respectfully chose to maintain the focus of the introduction on preoperative pain mechanisms relevant to the objectives of this study.

Comment 8: Minor language revisions are recommended, and project-specific terms such as “painCELL” and “painHS” may benefit from brief clarification.

Response 8: Thank you for this helpful suggestion. We agree that the project-specific terminology used in the manuscript may benefit from clarification for readers unfamiliar with these terms. We have therefore added brief explanatory descriptions when first introducing the terms “painCELL” and “painHS” in the Methods section. In brief, “painCELL” refers to the functional immune stimulation assay used to quantify cytokine release (IL-1β) from peripheral blood mononuclear cells (PBMCs) and synovial fluid mononuclear cells (SFMCs) following Toll-like receptor stimulation. “painHS” refers to the hyperspectral (HS) imaging-based analysis of immune cells used to characterise cellular autofluorescence signatures and identify potential phenotypic differences between participant groups.

Minor language edits were also performed throughout the manuscript to improve clarity and readability.

Line 59, deleted “(painCELL)” and “(painHS)”

Line 139-140, added: “(…)CSK4 (PAM3, a synthetic lipopeptide which functions as an agonist for TLR-2)”

Line 140-141, added “(…) lipopolysaccharide (LPS, i.e., endotoxin, gram-negative bacterial cell wall component which functions as an agonist for TLR-4) (…)”

Line 279-280, added: “(…) functional immune stimulation assay used to quantify cytokine release (IL-1β) (i.e., “painCELL test”) (…)”

Line 280-283, added “(…) hyperspectral (HS) imaging-based analysis of immune cells used to characterise cellular autofluorescence signatures and identify potential phenotypic differences between participant groups (i.e., “painHS test”), (…)”

Line 322, deleted “Toll-like receptor 2 (“ and “(…)CSK4” and added “-“ between TLR and 2.

Line 323, deleted “(…) lipopolysaccharide((…)“ and added “-“ between TLR and 4.

Line 396, added “-“ between TLR and 2.

General language edits:

Line 95, delete “(OA)”

Line 129, delete “(OA)” and inserted “osteoarthritis”

Line 130, delete “(OA)” and inserted “osteoarthritis”

Line 138, delete “(OA)” and inserted “osteoarthritis”

Line 206, delete “(OA)”

Line 210, delete “(OA)”

Line 228, delete “(OA)” and inserted “osteoarthritis”

Line 232, delete “(OA)” and inserted “osteoarthritis”

Line 233, delete “(OA)” and inserted “osteoarthritis”

Line 251, delete “(OA)” and inserted “osteoarthritis”

Line 406, delete “(OA)” and inserted “osteoarthritis”

Line 414, delete “(OA)” and inserted “osteoarthritis”

Line 447, delete “(OA)” and inserted “osteoarthritis”

Line 448, delete “(OA)” and inserted “osteoarthritis”

References

1. Evans SF, Kwok YH, Solterbeck A, Liu J, Hutchinson MR, Hull ML, Rolan PE (2020) Toll-Like Receptor Responsiveness of Peripheral Blood Mononuclear Cells in Young Women with Dysmenorrhea. J Pain Res 13:503–516

2. Crijns TJ, Brinkman N, Ramtin S, Ring D, Doornberg J, Jutte P, Koenig K (2022) Are There Distinct Statistical Groupings of Mental Health Factors and Pathophysiology Severity Among People with Hip and Knee Osteoarthritis Presenting for Specialty Care? Clin Orthop Relat Res 480(2):298–309

3. Innmann MM, Lunz A, Fröhlich L, Bruckner T, Merle C, Reiner T, Schiltenwolf M (2023) What Is the Correlation between Clinical and Radiographic Findings in Patients with Advanced Osteoarthritis of the Knee? J Clin MedDOI: 10.3390/jcm12165420

4. Sonobe T, Otani K, Sekiguchi M, Otoshi K, Nikaido T, Konno S, Matsumoto Y (2024) Influence of Knee Osteoarthritis Severity, Knee Pain, and Depression on Physical Function: A Cross-Sectional Study. Clin Interv Aging 19(October 2024):1653–1662

5. Eberly L, Richter D, Comerci G, Ocksrider J, Mercer D, Mlady G, Wascher D, Schenck R (2018) Psychosocial and demographic factors influencing pain scores of patients with knee osteoarthritis. Isales CM (ed) PLoS One 13(4):e0195075

6. Hilibrand AS, Farid AR, Desai V, Lange JK, Shah VM, Chen AF (2025) The Relationship Between Radiographic Osteoarthritis, Step Counts, and Pain Levels in Patients Undergoing Hip and Knee Arthroplasty. J Am Acad Orthop Surg Glob Res RevDOI: 10.5435/JAAOSGlobal-D-24-00385

7. Aalders MB, van der List JP, Keijser LCM, Benner JL. Anxiety and depression prior to total knee arthroplasty are associated with worse pain and subjective function: A prospective comparative study. Knee Surg Sports Traumatol Arthrosc. 2025 Jan;33(1):308-318. doi: 10.1002/ksa.12336. Epub 2024 Jun 29. PMID: 38943459; PMCID: PMC11716335.

8. van der List JP, Benner JL, Temmerman OPP, Keijser LCM. Preoperative Pain Catastrophizing Prior to Total Knee Arthroplasty is Associated With Worse Preoperative Symptoms and More Clinical Improvement: A Prospective Comparative Study. J Arthroplasty. 2023 Mar;38(3):470-475. doi: 10.1016/j.arth.2022.09.016. Epub 2022 Sep 17. PMID: 36126888.

VERSION 2 - REVIEW

**Reviewer**	**2**
**Name**	**Ramadanov, Nikolai**
**Affiliation**	**University Hospital Brandenburg/Havel Brandenburg Medical School**
**Date**	**02-Apr-2026**
**COI**	

This is a clinically interesting and potentially valuable translational study protocol addressing an important gap in the objective assessment of pain in knee osteoarthritis. However, in its current form, the protocol remains somewhat overambitious for the planned sample size, and the analytical strategy would benefit from clearer prioritization. In particular, the primary endpoint and primary hypothesis should be defined more explicitly, and the manuscript should more clearly frame the study as exploratory/hypothesis-generating rather than implying biomarker validation.

VERSION 2 - AUTHOR RESPONSE

Reviewer: 2

Dr. Nikolai Ramadanov, University Hospital Brandenburg/Havel Brandenburg Medical School, Brandenburg Medical School Theodor Fontane
Comments to the Author:
This is a clinically interesting and potentially valuable translational study protocol addressing an important gap in the objective assessment of pain in knee osteoarthritis. However, in its current form, the protocol remains somewhat overambitious for the planned sample size, and the analytical strategy would benefit from clearer prioritization. In particular, the primary endpoint and primary hypothesis should be defined more explicitly, and the manuscript should more clearly frame the study as exploratory/hypothesis-generating rather than implying biomarker validation.

Reviewer: 2
If you have selected 'Yes' above, please provide details of any competing interests.: Not applicable

Comment 1: in its current form, the protocol remains somewhat overambitious for the planned sample size

Response 1:
We thank the reviewer for this important comment. We agree that the scope of the study should be clearly aligned with its sample size and intended purpose.
The present study is designed as an initial exploratory, hypothesis-generating study in an orthopaedic population, aimed at identifying potential associations between peripheral immune responses and pain. The sample size was determined based on a formal a priori power calculation for the primary outcome (EC50), and exceeds the number required for this primary comparison. The study design, including the sample size calculation and overall analytical framework, was developed in close collaboration with senior clinical and translational research experts in this field (professor Mark Hutchinson and professor Rudi Jaarsma). Following the reviewer's comment, this has been re-evaluated within the study expert team, and we remain confident that the planned sample size is appropriate for the primary analyses and exploratory aims of the study. We acknowledge that the broader analytical framework, including secondary and exploratory analyses, may appear extensive relative to the sample size. These analyses are not intended for definitive inference or validation, but rather to explore potential multidimensional relationships and generate

hypotheses for future, larger-scale studies. Furthermore, we agree that the previous wording of the manuscript was insufficient in addressing this issue. Therefore, to address this comment, we have revised the manuscript to 1) clearly define the primary endpoint and primary hypothesis, 2) explicitly distinguish primary from secondary/exploratory analyses, and 3) consistently frame the study as exploratory rather than confirmatory.

Comment 2: the analytical strategy would benefit from clearer prioritization. In particular, the primary endpoint and primary hypothesis should be defined more explicitly.

Response 2:
Thank you very much for this valuable suggestion. We agree that clearer prioritisation of the analytical strategy improves the readability and scientific focus of the protocol. We have now explicitly defined the primary aim and hypothesis, and primary endpoint, in the abstract, introduction, and methods section. The primary analysis focuses on differences in peripheral immune response parameters (EC50 for IL-1β) between painful osteoarthritic knees and pain-free controls. All additional analyses, including associations with pain and function PROMs and psychological factors, and complementary characterising immune cell phenotypes through hyperspectral imaging, are now explicitly described as secondary and exploratory.

Comment 3: the manuscript should more clearly frame the study as exploratory/hypothesis-generating rather than implying biomarker validation.

Response 3:
We thank the reviewer for this important remark and fully agree. Indeed, the study should be clearly positioned as exploratory rather than implying biomarker validation. The aim of this study is not to validate pain biomarkers, but to explore whether peripheral immune responses show potential as candidate biomarkers in an orthopaedic population. Therefore, we have revised the manuscript throughout to reflect this, including 1) replacing terminology suggesting "validation" with "exploratory" language, 2) emphasising the hypothesis-generating nature of the study, and 3) clarifying that findings will serve as a basis for future validation studies. We believe this improved the framing of the study more accurately, and reflects the study's intended contribution.

Changes made:

Abstract

Line 44: change “investigate” to “explore”

Line 45: insert “(interleukin-1 beta, IL-1β) are associated with pain and may” and delete “can”

Line 47: insert “pain and psychological (“

Line 48: change “catatrophising” into “catastrophizing”. Addition of “PROMs”.

Line 56: change “mental health” into “psychological”.

Line 60: change “interleukin-1 beta” into “IL-1β”.

Introduction

Line 131-151: changed last Alinea into: “The primary aim of this exploratory study is to investigate whether peripheral immune responses, IL-1β expression quantified from both peripheral blood mononuclear cells (PBMCs) and synovial fluid mononuclear cells (SFMCs), differ between patients with symptomatic knee osteoarthritis awaiting TKA and pain-free healthy controls. We hypothesize that stimulated immune responses (IL-1β release) are elevated in patients with symptomatic knee osteoarthritis compared to pain-free controls. Secondary exploratory analyses will 1) examine associations between immune responses, subjective pain measures, and psychological factors, and 2) evaluate hyperspectral imaging as a complementary approach for characterising immune cell phenotypes [17]. This study is intended as a hypothesis-generating study to inform future research on objective pain biomarkers in orthopaedic populations, and to address the absence of objective, biologically anchored pain measurement in orthopaedic surgery. This exploratory study applies neuroimmune biomarker technology to musculoskeletal pain, and may provide a foundation for integrating objective biological markers with subjective pain assessment to improve clinical decision-making and ultimately contribute to a more multidimensional approach of pain assessment.”

Methods and analysis

Line 192: added “an exploratory”

Line 210-224: deleted “The primary aim is to establish a novel framework for identifying and validating pain biomarkers in orthopaedic patients undergoing TKA, with the specific goal of augmenting subjective pain scales with precise, objective, biomarker-based methods for pain assessment and clinical decision-making. This will be conducted through correlating pain

biomarkers (peripheral immune responses) with subjective pain levels in TKA patients. Furthermore, the relationship between hyperspectral imaging hues and pain markers will be examined. Lastly, pain marker elevation in patients with anxiety, depression, or catastrophizing tendencies will be assessed."

And added: "The primary aim of this exploratory study is to assess whether peripheral immune response parameters (IL-1β release following TLR-2 and TLR-4 activation) differ between painful osteoarthritic knees and pain-free controls. This will be conducted through measuring peripheral immune responses (pain biomarkers, i.e., IL-1β expression), quantified from both peripheral blood mononuclear cells (PBMCs) and synovial fluid mononuclear cells (SFMCs) following TLR-2 and TLR-4 activation using PAM3CSK4 (PAM3, a synthetic lipopeptide which functions as an agonist for TLR-2) and lipopolysaccharide (LPS, i.e., endotoxin, gram-negative bacterial cell wall component which functions as an agonist for TLR-4), between patients with symptomatic knee osteoarthritis awaiting TKA and pain-free healthy controls.

Secondary exploratory aims include 1) assessing associations between immune responses (IL-1β expression) and subjective pain scores, 2) exploring relationships between immune responses and psychological factors (anxiety, depression, pain catastrophizing), and 3) evaluating hyperspectral imaging as a complementary approach for characterising immune cell phenotypes by assessing the relationship between hyperspectral imaging hues and IL-1β expression."

Line 303: added "The primary outcome included EC50 (concentration-response parameter: concentration which produces 50% of the maximal effect) for IL-1β release, to determine the responsiveness of PBMC and SFMC to LPS and PAM3 stimulation."

Line 305: deleted "The o", into: "Osteoarthritis" and added "severity".

Line 426-434: deleted underline of text.

Line 432-434: changed "catastrophising" into "catastrophizing", and added "All secondary analyses, including multivariable and machine-learning approaches, are exploratory in nature and aimed at hypothesis generation rather than confirmatory inference."

VERSION 3 - REVIEW

Reviewer 2

**Name**	**Ramadanov, Nikolai**
**Affiliation**	**University Hospital Brandenburg/Havel Brandenburg Medical School**
**Date**	**06-May-2026**
**COI**	

Thanks for considering my suggestions.
